# Widefield SS-OCTA-detected retinal vascular changes and their correlation with DME in diabetes

**DOI:** 10.3389/fendo.2026.1858145

**Published:** 2026-06-08

**Authors:** Chuanzhen Zheng, Yang Liu, Ruolan Ling, Miao Liu, Wenting Sun, Shiya Tang, Chuntao Lei, Huaqin Xia, Yong Zeng

**Affiliations:** 1 Department of Ophthalmology, Sichuan Provincial People’s Hospital, School of Medicine, University of Electronic Science and Technology of China, Chengdu, China; 2 Department of School Hospital, Southwestern University of Finance and Economics, Chengdu, China; 3 Department of Ophthalmology, Chengdu Wenjiang District People’s Hospital, Chengdu, China

**Keywords:** deep vascular complex, diabetic macular edema, optical coherence tomography angiography, superficial vascular complex, vascular density, widefield

## Abstract

**Purpose:**

This study investigated retinal blood flow alterations in diabetic macular edema (DME) using ultra-widefield optical coherence tomography angiography (OCTA).

**Methods:**

We enrolled 31 healthy controls (NC, 56 eyes), 40 diabetes patients without diabetic retinopathy (DM, 79 eyes), 25 non-proliferative diabetic retinopathy patients (NPDR, 47 eyes), and 42 DME patients (DME, 54 eyes). OCTA images were analyzed using five-quadrant and concentric zonal divisions to assess vascular density (VD) in the superficial (SVC) and deep vascular complexes (DVC).

**Results:**

In the DM group, nasal SVC VD was significantly reduced in the N11, N16, and N21 regions. NPDR patients showed widespread SVC VD reduction compared to controls and DM patients, while DME patients exhibited elevated SVC VD in the peripheral retina (11–21 mm). DVC changes were significant only in the macular area in NPDR group, with DME patients showing increased VD in all regions except I21. Peripheral DVC VD (T11, T16, S16) strongly correlated with DME on ROC analysis.

**Conclusions:**

Nasal SVC vascular alterations are among the detectable early intergroup differences in preclinical DR, and peripheral DVC VD is closely correlated with the presence of DME.

## 1 Introduction

Diabetic retinopathy (DR), a principal ocular complication of diabetes mellitus (DM), represents a leading cause of severe visual impairment and blindness worldwide (1). As the disease progresses, the retina may develop microaneurysms, hemorrhages, hard exudates, cotton wool spots, retinal neovascularization, fibrovascular proliferation, vitreous hemorrhage, tractional retinal detachment, or neovascular glaucoma, ultimately resulting in blindness (2). Once DR begins, patients must undergo long-term follow-up with a retinal specialist to monitor disease progression and receive timely intervention when necessary to prevent irreversible vision loss (2). Diabetic macular edema (DME), a critical complication of DR, can occur at any stage of the disease, primarily affects the macula and can cause significant vision impairment (3). Early detection and intervention can greatly preserve patients’ vision. In recent years, the widespread use of intravitreal anti-VEGF drugs has enabled most patients to receive treatment at a low cost (4).

Optical coherence tomography angiography (OCTA) is a rapid, *in vivo*, non-invasive imaging technique. Unlike traditional fluorescein angiography (FA), OCTA enables detailed visualization of retinal and choroidal microvasculature without the need for contrast agents (5). Current swept-source optical coherence tomography angiography (SS-OCTA) achieves a 6mm scanning depth, 24mm scan length, 24mm×20mm fundus blood flow OCTA, and structural OCT with an axial optical resolution of 3.8μm, making it highly sensitive to subtle vascular abnormalities that other imaging techniques may miss (6). Additionally, OCTA can quantitatively analyze key retinal features, such as the thickness of the layered retina, vascular density, non-perfusion area, and foveal avascular zone (FAZ) size, thereby providing valuable quantitative data on the microvascular health of the retina and choroid (7–9).

In the management of DME, OCTA serves as a crucial monitoring tool (10). Follow-up mode tomography can track the resolution of macular edema and evaluate the efficacy of drug treatments, while enface imaging monitors non-perfusion areas and neovascularization, providing timely guidance for laser intervention (11). In recent years, ultra-widefield SS-OCTA has enabled the assessment of blood flow information beyond the macular region, extending to the peripheral retina. Our research has expanded from the original 3×3mm and 6×6mm scans to 12×12mm and even 24×20mm scans (12). Consequently, the blood flow perfusion status of the peripheral retina under DME conditions, its correlation with macular edema, or its changes during DR progression represent pressing scientific questions our research team aims to explore.

OCTA has emerged as a pivotal non-invasive imaging tool for evaluating retinal and choroidal microvasculature in DR and macular edema. Its ability to provide high-resolution, quantitative vascular analysis and monitor disease progression offers significant clinical advantages over traditional techniques. The advent of ultra-widefield SS-OCTA further enables comprehensive assessment of peripheral retinal perfusion, opening new avenues for investigating the relationship between DME and peripheral vascular changes in DR progression. These advancements underscore OCTA’s critical role in early detection, treatment monitoring, and guiding therapeutic interventions for DR. This study will include patients with DM without DR, those with mild to moderate DR, and individuals with DME. SS-OCTA will be utilized to observe retinal blood flow changes and monitor the dynamic alterations in peripheral retinal perfusion during the progression of DR. The study aims to analyze the patterns of retinal blood flow changes during DR progression and explore potential biomarkers for early prevention and treatment of DR.

## 2 Methods

### 2.1 Study design, data sources, and study participants

This retrospective study enrolled 31 healthy controls (NC, 56 eyes), 40 DM patients (79 eyes), 25 NPDR patients (47 eyes), and 42 DME patients (54 eyes) who visited the Ophthalmology Department of Sichuan Provincial People’s Hospital from January 2022 to June 2025. The study protocol complied with the Declaration of Helsinki and received ethical approval from the Institutional Review Board of Sichuan Provincial People’s Hospital.

### 2.2 Inclusion and exclusion criteria

Inclusion criteria for the NC: (1) age ≥18 years; (2) signed informed consent with good compliance. Exclusion criteria for NC group: (1) media opacity; (2) coexisting macular pathologies including epiretinal membrane, macular hole, or macular edema secondary to retinal vein occlusion, uveitis, or central serous chorioretinopathy; (3) history of retinal laser photocoagulation or intravitreal injections; (4) glaucoma or ocular hypertension in either eye; (5) Renal failure, Behçet’s disease, systemic lupus erythematosus, and other systemic diseases that may affect vascular function. DM group inclusion followed the same criteria as NC group, with additional requirement of type 2 diabetes diagnosis per International Diabetes Federation guidelines (13). Patients in the NPDR group were diagnosed with non-proliferative diabetic retinopathy according to the Early Treatment Diabetic Retinopathy Study (ETDRS) classification standards and ruled out the presence of DME by OCT (2), primarily presenting with microaneurysms, retinal hemorrhages, hard exudates, and/or cotton wool spots, were included in the NPDR group. NPDR patients confirmed to have DME by OCT examination were included in the DME group.

### 2.3 Baseline data

Baseline demographic and clinical characteristics of all enrolled patients were systematically recorded, including age, gender, height, weight, medical history, fasting blood glucose (FBG), glycated hemoglobin (HbA1c), axial length (AL), intraocular pressure (IOP), and best-corrected visual acuity (BCVA). Comprehensive ophthalmic examinations were performed using standardized protocols, including detailed slit-lamp microscopy to document anterior and posterior segment findings. Fundus imaging was obtained for all participants using the Daytona ultra-widefield retinal imaging system (Optos, UK). The diagnosis of DR was independently confirmed by two experienced retinal specialists through masked evaluation of fundus photographs.

### 2.4 OCT and OCTA data

The OCT/OCTA device used in this study was provided by TowardPi Medical Technology, Beijing, China. The TowardPi OCT features a 6mm scanning depth, 3.8μm axial optical resolution, and a high-penetration wavelength of 1060 nm. Only images with a quality score ≥8 were included for further analysis. The raw retinal blood flow data were obtained and exported using the device’s built-in analysis software for subsequent evaluation. Using the device’s proprietary software, the retina was divided into four fan-shaped regions centered on the macula: superior (S), inferior (I), nasal (N), and temporal (T). Additionally, OCTA images were segmented into a central macular (CM) zone with a diameter of 1 mm, as well as concentric annular regions at distances of 1–3 mm (S/I/N/T 3 zone), 3–6 mm (S/I/N/T 6 zone), 6–11 mm (S/I/N/T 11 zone), 11–16 mm (S/I/N/T 16 zone), and 16–21 mm (S/I/N/T 21 zone). We compared the average vessel density (VD) of the superficial vascular complex (SVC) and deep vascular complex (DVC) across different quadrants and concentric annular distances (14).

### 2.5 Statistical analysis

Statistical analysis was conducted utilizing SPSS Statistics software. Measurements of BCVA were converted into the LogMAR for enhanced statistical evaluation. Data normality was evaluated using the Kolmogorov–Smirnov test. For continuous variables adhering to a normal distribution, descriptive statistics were represented by the mean and standard deviation (SD), while for those not normally distributed, the median and interquartile range (IQR) were employed. The comparisons of VD across multiple groups were analyzed using a two-way analysis of variance (ANOVA). For *post-hoc* pairwise comparisons, Tukey’s honestly significant difference (HSD) test was employed. Categorical variables were presented as numbers and percentages. Patient characteristics and baseline data were compared between groups using an independent t-test, one-way ANOVA, or Mann-Whitney U test. Chi-square test or Fisher’s exact test was used to assess the significance of frequencies. A P < 0.05 was considered significant.

## 3 Results

### 3.1 Baseline characteristics

The study cohort comprised 56 eyes of NC, 79 eyes with DM, 47 eyes with NPDR, and 54 eyes with DME. all age- and gender-matched. **Table 1** presents the baseline demographic and clinical characteristics across the four study groups. No statistically significant differences were found among groups regarding gender distribution (p=0.407), mean age (p=0.087), axial length (p=0.709), incidence of hypertension (0.358) or intraocular pressure (p=0.606). However, best-corrected visual acuity (BCVA) differed significantly among groups (p<0.001). Among the diabetic subgroups (DM, NPDR, DME), FBG and HbA1c levels showed no significant variation (p=0.085 and 0.102).

**Table 1:** Baseline features of patients and healthy controls.

Characteristic	NC	DM	NPDR	DME	P Values
Patients (Female)	31(18)	40(18)	25(11)	42(16)	0.407
Ages	56.00 ± 7.52	58.32 ± 10.53	53.88 ± 7.96	57.70 ± 10.05	0.087
Eyes	56	79	47	54	N/A
HBP	31(13)	40(24)	25(11)	42(23)	0.358
Type of DM	N/A	2	2	2	N/A
FBG	N/A	7.91 ± 2.40	9.24 ± 3.75	7.78 ± 2.19	0.085
HbA1c	N/A	7.54 ± 1.51	8.25 ± 0.90	7.45 ± 1.77	0.102
AL	23.31 ± 0.67	23.56 ± 0.72	23.46 ± 1.10	23.46 ± 0.86	0.709
IOP	14.34 ± 2.27	14.26 ± 3.10	14.84 ± 2.44	14.59 ± 2.73	0.606
BCVA (Logmar)	0.007 ± 0.037	0.035 ± 0.074	0.121 ± 0.161	0.195 ± 0.303	<0.001

Values are shown as means ± SD. NC, Normal Control; HBP, high blood pressure; DM, diabetes mellitus; NPDR, non-proliferative diabetic retinopathy; DME, diabetic macular edema; FBG, fasting blood-glucose; HbA1c, Hemoglobin A1C; AL, axial lengths; IOP, intra-ocular pressure; BCVA, best correct visual acuity; A P value less than 0.05 was considered statistically significant.

### 3.2 SVC analysis

**Figure 1:**
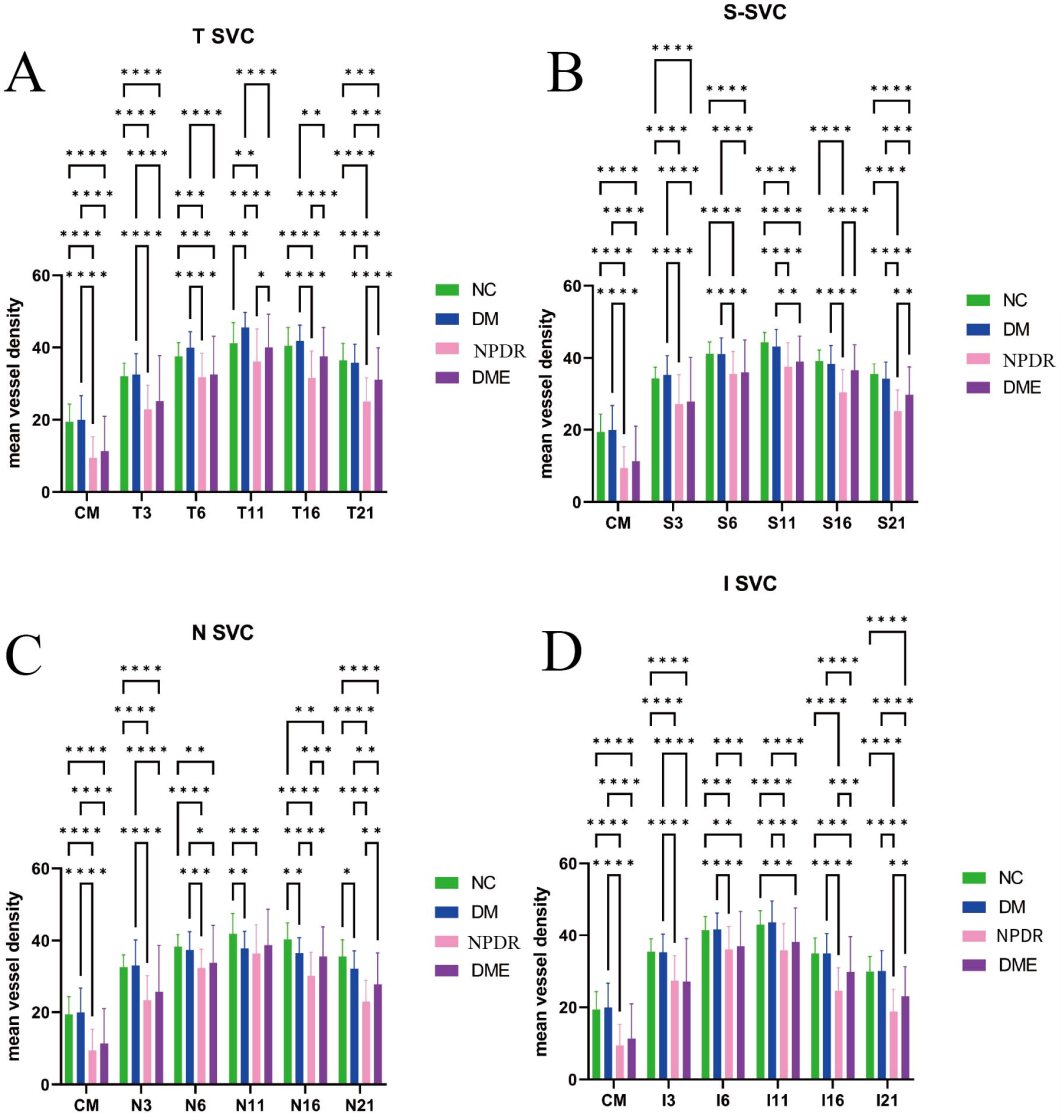
Comparisons of SVC VD between the NC, DM, NPDR and DME of each quadrant. **(A)** Temporal quadrant, **(B)** Superior quadrant, **(C)** Nasal quadrant, and **(D)** Inferior quadrant. SVC, superficial vascular complex; VD, vascular density; NC, normal control; DM, diabetes mellitus; NPDR, non-proliferative diabetic retinopathy; DME, diabetic macular edema. *P < 0.05, **P < 0.01, ***P < 0.001, ****P < 0.0001.

Compared to NC group, the DM group exhibited significant VD reduction in the nasal SVC at N11, N16, and N21 regions. The NPDR group demonstrated significantly decreased SVC VD across all quadrants (temporal, superior, nasal, inferior), except at N11. When comparing DME and NPDR groups, SVC VD showed significant increases in multiple regions (T11, T16, T21, S16, S21, N16, N21, I16, I21). However, compared to the DM group, the DME group maintained significantly lower VD values in most regions except S16, N11, and N16. Complete regional comparisons are illustrated in **Figure 1**, **Table 2**.

**Table 2:** **Figure 1** related data.

Region	NC	DM	NPDR	DME	p1 (NC vs DM)	p2 (DM vs NPDR)	p3 (DM vs DME)	p4 (NPDR vs DME)
CM	19.36 ± 4.97	19.94 ± 6.73	9.4 ± 5.89	11.33 ± 9.63	0.9623	<0.0001	<0.0001	0.4901
T3	32.02 ± 3.66	32.54 ± 5.8	22.85 ± 6.7	25.17 ± 12.63	0.9713	<0.0001	<0.0001	0.3247
T6	37.57 ± 3.82	39.97 ± 4.39	31.83 ± 6.58	32.52 ± 10.6	0.1835	<0.0001	<0.0001	0.9579
T11	41.23 ± 5.62	45.56 ± 4.16	36.15 ± 8.97	40.04 ± 9.18	0.0017	<0.0001	<0.0001	0.0228
T16	40.48 ± 5.02	41.84 ± 4.37	31.6 ± 7.42	37.56 ± 7.96	0.6687	<0.0001	0.0023	<0.0001
T21	36.45 ± 4.7	35.85 ± 5.1	25.02 ± 6.61	31.02 ± 8.92	0.9588	<0.0001	0.0004	<0.0001
S3	34.2 ± 3.07	35.15 ± 5.47	27.15 ± 8.09	27.76 ± 12.4	0.8199	<0.0001	<0.0001	0.962
S6	41.2 ± 3.2	41.08 ± 4.5	35.45 ± 6.37	35.87 ± 9.08	0.9995	<0.0001	<0.0001	0.9867
S11	44.36 ± 2.71	43.14 ± 4.77	37.36 ± 6.86	39.04 ± 7.02	0.6833	<0.0001	0.0013	0.5393
S16	39.14 ± 3.05	38.23 ± 5.19	30.32 ± 6.32	36.46 ± 7.21	0.8383	<0.0001	0.3837	<0.0001
S21	35.41 ± 2.92	34.13 ± 4.76	25.13 ± 5.87	29.69 ± 7.68	0.6456	<0.0001	0.0004	0.0016
N3	32.64 ± 3.4	33.06 ± 7.09	23.3 ± 6.98	25.65 ± 13.04	0.9851	<0.0001	<0.0001	0.3134
N6	38.36 ± 3.29	37.41 ± 5.06	32.4 ± 5.21	33.87 ± 10.35	0.8564	0.0004	0.0185	0.706
N11	41.91 ± 5.59	37.86 ± 4.73	36.43 ± 7.96	38.76 ± 9.93	0.0041	0.6663	0.8796	0.3197
N16	40.34 ± 4.6	36.56 ± 4.26	30.3 ± 6.46	35.63 ± 8.21	0.0087	<0.0001	0.8694	0.0006
N21	35.61 ± 4.63	32.24 ± 4.94	22.91 ± 6.04	27.78 ± 8.81	0.0255	<0.0001	0.0013	0.0022
I3	35.38 ± 3.62	35.23 ± 5.19	27.38 ± 6.89	27.15 ± 11.89	0.9993	<0.0001	<0.0001	0.4796
I6	41.52 ± 3.78	41.66 ± 4.63	36.04 ± 6.5	36.89 ± 9.76	0.9994	<0.0001	<0.0001	0.9981
I11	43.07 ± 3.82	43.67 ± 5.95	35.79 ± 7.5	38.11 ± 9.58	0.9572	<0.0001	0.0004	0.923
I16	34.93 ± 4.23	34.95 ± 5.64	24.6 ± 6.33	29.78 ± 9.81	0.9999	<0.0001	<0.0001	0.311
I21	29.89 ± 4.15	30.06 ± 5.63	18.81 ± 6.16	23.04 ± 8.18	1.00	<0.0001	<0.0001	0.0007

### 3.3 DVC analysis

**Figure 2:**
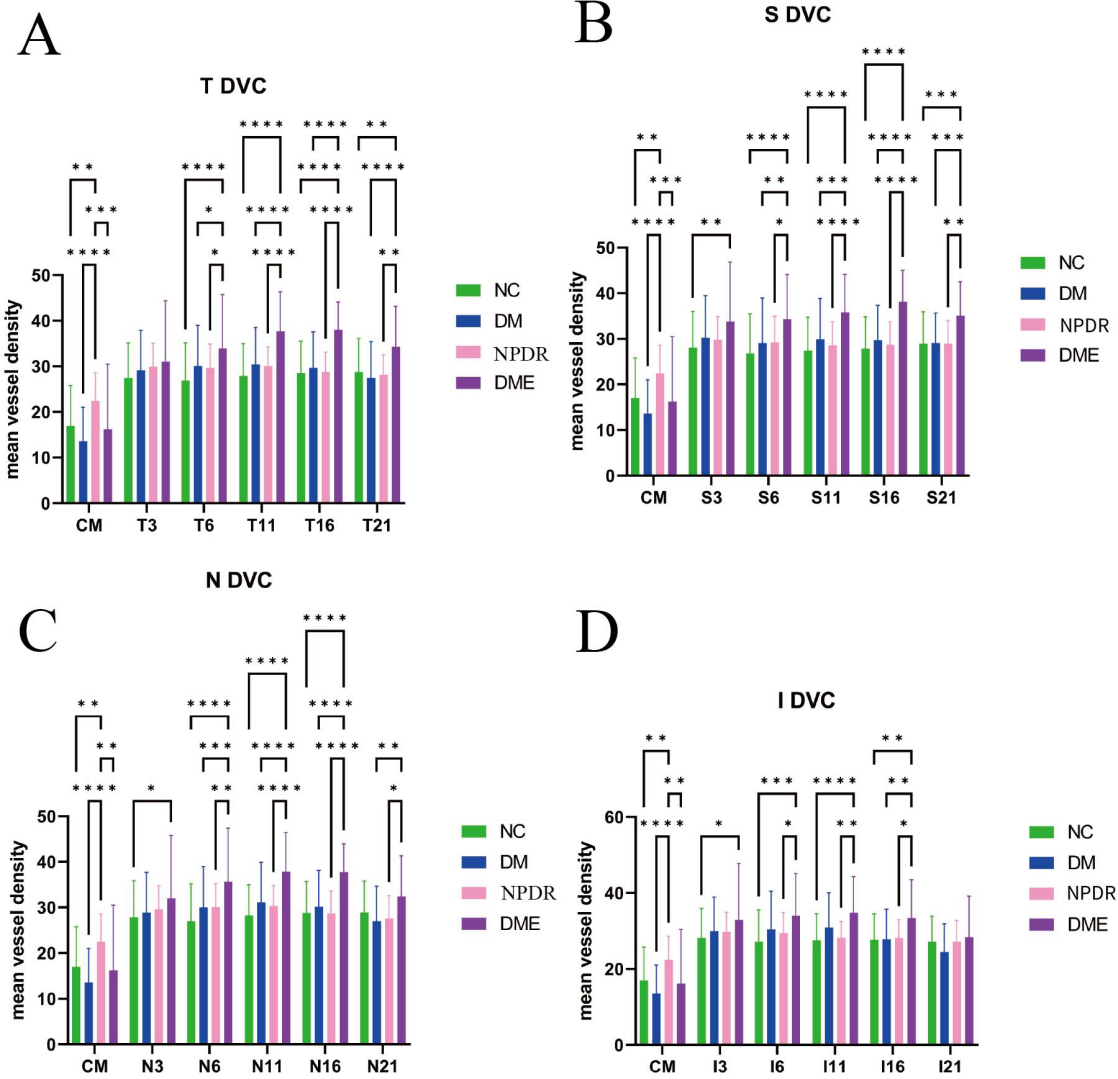
Comparisons of DVC VD between the NC, DM, NPDR and DME of each quadrant. **(A)** Temporal quadrant, **(B)** Superior quadrant, **(C)** Nasal quadrant, and **(D)** Inferior quadrant. SVC, superficial vascular complex; VD, vascular density; NC, normal control; DM, diabetes mellitus; NPDR, non-proliferative diabetic retinopathy; DME, diabetic macular edema. *P < 0.05, **P < 0.01, ***P < 0.001, ****P < 0.0001.

Macular DVC VD showed a non-significant decreasing trend in DM patients versus controls. The NPDR group displayed significantly higher VD than the DM group, while the DME group showed significant reduction compared to NPDR. In extramacular regions, no significant VD differences were observed between NPDR and DM groups. However, the DME group exhibited significantly increased VD versus NPDR in multiple regions (T11, T16, T21, S6, S11, S16, S21, N6, N11, N16, N21, I6, I11, I16). Complete data are presented in **Figure 2**, **Table 3**.

**Table 3:** **Figure 2** related data.

Region	NC	DM	NPDR	DME	p1 (DM vs NPDR)	p2 (DM vs DME)	p3 (NPDR vs DME)
CM	16.96 ± 8.79	13.58 ± 7.45	22.43 ± 6.18	16.17 ± 14.33	<0.0001	0.2919	0.0009
T3	27.45 ± 7.7	29.16 ± 8.73	29.96 ± 5.09	31.02 ± 13.37	0.9547	0.5857	0.9188
T6	26.88 ± 8.27	30.08 ± 8.95	29.66 ± 5.26	33.94 ± 11.79	0.9929	0.0418	0.0481
T11	27.89 ± 7.09	30.38 ± 8.14	30 ± 4.27	37.69 ± 8.66	0.9946	<0.0001	<0.0001
T16	28.46 ± 7.06	29.65 ± 7.94	28.83 ± 4.27	38.02 ± 6.1	0.9509	<0.0001	<0.0001
T21	28.75 ± 7.42	27.43 ± 7.98	28.11 ± 4.37	34.31 ± 8.85	0.9712	<0.0001	0.0011
S3	28.09 ± 7.93	30.25 ± 9.2	29.83 ± 5.06	33.78 ± 13.08	0.9925	0.0746	0.0784
S6	26.82 ± 8.68	29.11 ± 9.85	29.21 ± 5.73	34.31 ± 9.77	>0.9999	0.0021	0.0108
S11	27.39 ± 7.36	29.91 ± 8.95	28.57 ± 5.2	35.81 ± 8.33	0.8161	0.0003	<0.0001
S16	27.86 ± 7	29.7 ± 7.65	28.74 ± 4.97	38.11 ± 6.93	0.924	<0.0001	<0.0001
S21	28.95 ± 6.98	29.09 ± 6.56	28.96 ± 5.02	35.07 ± 7.46	0.9998	0.0003	0.0012
N3	27.84 ± 8.01	28.86 ± 8.83	29.6 ± 5.17	32 ± 13.78	0.9641	0.1452	0.4737
N6	26.96 ± 8.24	30.03 ± 8.97	30.09 ± 5.18	35.65 ± 11.76	>0.9999	0.0008	0.0048
N11	28.25 ± 6.73	31.09 ± 8.8	30.3 ± 4.52	37.87 ± 8.58	0.9559	<0.0001	<0.0001
N16	28.82 ± 6.85	30.13 ± 8.03	28.74 ± 4.86	37.74 ± 6.18	0.8063	<0.0001	<0.0001
N21	28.89 ± 6.88	26.97 ± 7.65	27.57 ± 5.02	32.44 ± 8.88	0.98	0.0013	0.0187
I3	28.16 ± 7.77	29.96 ± 8.97	29.7 ± 5.2	32.89 ± 14.9	0.9985	0.2307	0.2615
I6	27.16 ± 8.4	30.43 ± 10.04	29.4 ± 5.48	34.02 ± 11.13	0.9202	0.0934	0.0412
I11	27.57 ± 6.96	30.92 ± 9.17	28.23 ± 4.28	34.8 ± 9.49	0.3405	0.0593	0.001
I16	27.7 ± 6.78	27.85 ± 7.86	28.19 ± 4.81	33.35 ± 10.1	0.9966	0.0021	0.0167
I21	27.2 ± 6.74	24.48 ± 7.38	27.17 ± 5.53	28.35 ± 10.83	0.3408	0.0594	0.9058

Values are shown as means ± SD.NC, normal control; DM, diabetes mellitus; NPDR, non-proliferative diabetic retinopathy; DME, diabetic macular edema.

### 3.4 Correlation between SVC VD and DME

**Figure 3:**
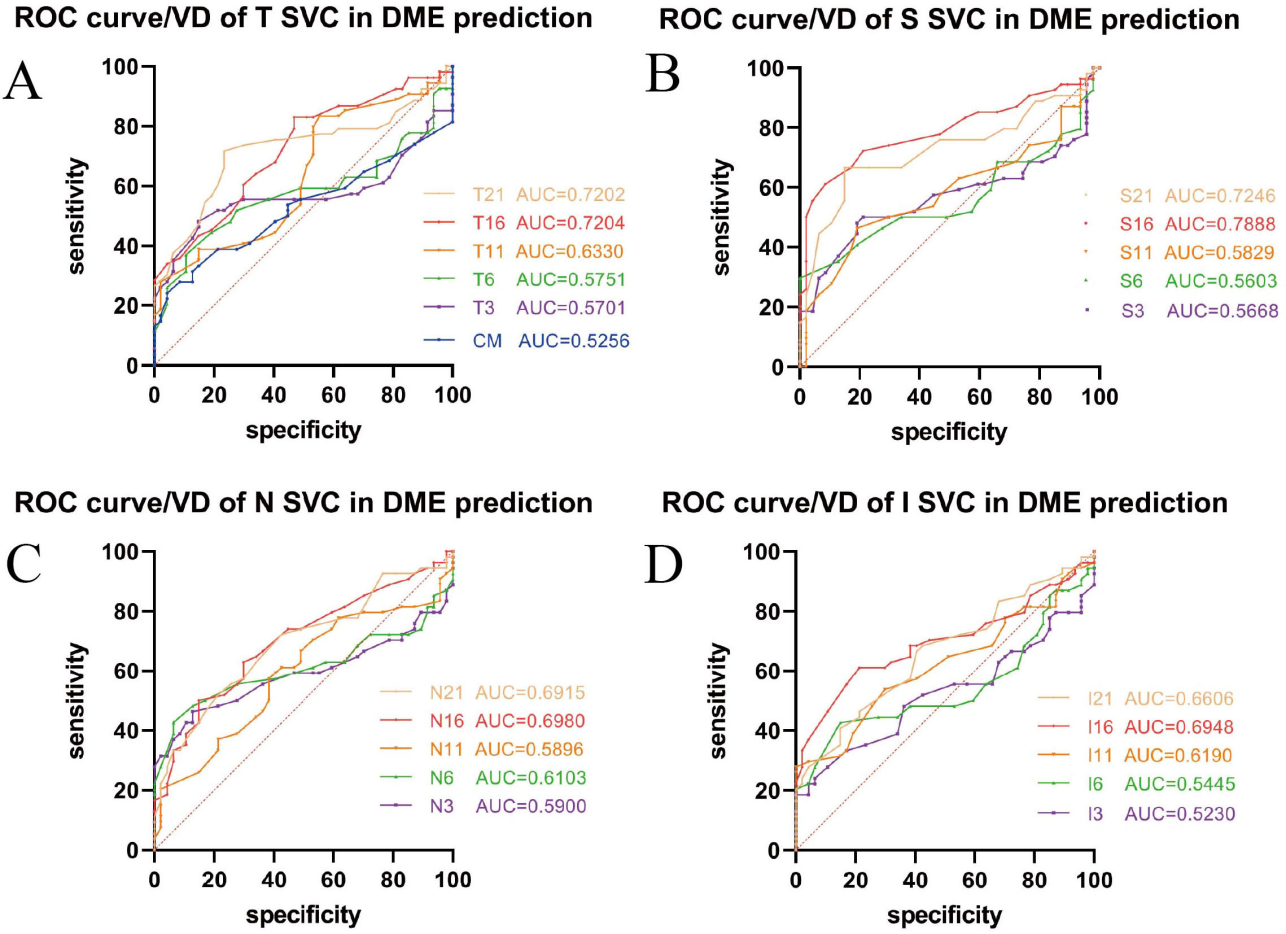
ROC curve analysis of SVC VD in patients with NPDR and DME. **(A)** Temporal quadrant, **(B)** Superior quadrant, **(C)** Nasal quadrant, and **(D)** Inferior quadrant. ROC, receiver operating characteristic; SVC, superficial vascular complex; VD, vascular density; NPDR, non-proliferative diabetic retinopathy; DME, diabetic macular edema.

Receiver operating characteristic (ROC) analysis demonstrated weak predictive value (AUC<0.7) for DME in nasal and inferior retinal regions. Regions T16, T21, S16, and S21 showed moderate predictive value (AUC>0.7), suggesting their potential association with DME pathogenesis. Complete ROC results are shown in **Figure 3**, **Table 4**.

**Table 4:** ROC curves for discriminating NPDR and DME.

Region	ROC curve/VD of T SVC in DME prediction		ROC curve/VD of T DVC in DME prediction	
	AUC	95% CI	AUC	95% CI
CM	0.5256	0.4106 - 0.6406	0.6584	0.5463 - 0.7705
T3	0.5701	0.4519 - 0.6883	0.5733	0.4526 - 0.6940
T6	0.5751	0.4603 - 0.6898	0.6923	0.5797 - 0.8049
T11	0.633	0.5241 - 0.7419	0.8314	0.7422 - 0.9206
T16	0.7204	0.6215 - 0.8193	0.8816	0.8082 - 0.9550
T21	0.7202	0.6170 - 0.8233	0.7618	0.6600 - 0.8636
I3	0.523	0.4085 - 0.6376	0.6243	0.5068 - 0.7418
I6	0.5445	0.4288 - 0.6602	0.71	0.6011 - 0.8189
I11	0.619	0.5094 - 0.7286	0.7541	0.6490 - 0.8593
I16	0.6948	0.5906 - 0.7991	0.727	0.6215 - 0.8324
I21	0.6606	0.5552 - 0.7659	0.5386	0.4221 - 0.6551
N3	0.59	0.4744 - 0.7057	0.6044	0.4840 - 0.7248
N6	0.6103	0.4954 - 0.7253	0.7118	0.6033 - 0.8203
N11	0.5896	0.4778 - 0.7015	0.7732	0.6754 - 0.8711
N16	0.698	0.5960 - 0.8000	0.8538	0.7749 - 0.9327
N21	0.6915	0.5888 - 0.7941	0.7374	0.6325 - 0.8423
S3	0.5668	0.4510 - 0.6826	0.6348	0.5139 - 0.7556
S6	0.5603	0.4452 - 0.6753	0.6852	0.5729 - 0.7974
S11	0.5829	0.4702 - 0.6957	0.7815	0.6850 - 0.8781
S16	0.7888	0.6979 - 0.8797	0.8544	0.7738 - 0.9350
S21	0.7246	0.6232 - 0.8260	0.75	0.6512 - 0.8488

DME, diabetic macular edema; NPDR, non-proliferative diabetic retinopathy; VD, vascular density; SVC, superficial vascular complex; DVC, deep vascular complex.

### 3.5 Correlation between DVC VD and DME

**Figure 4:**
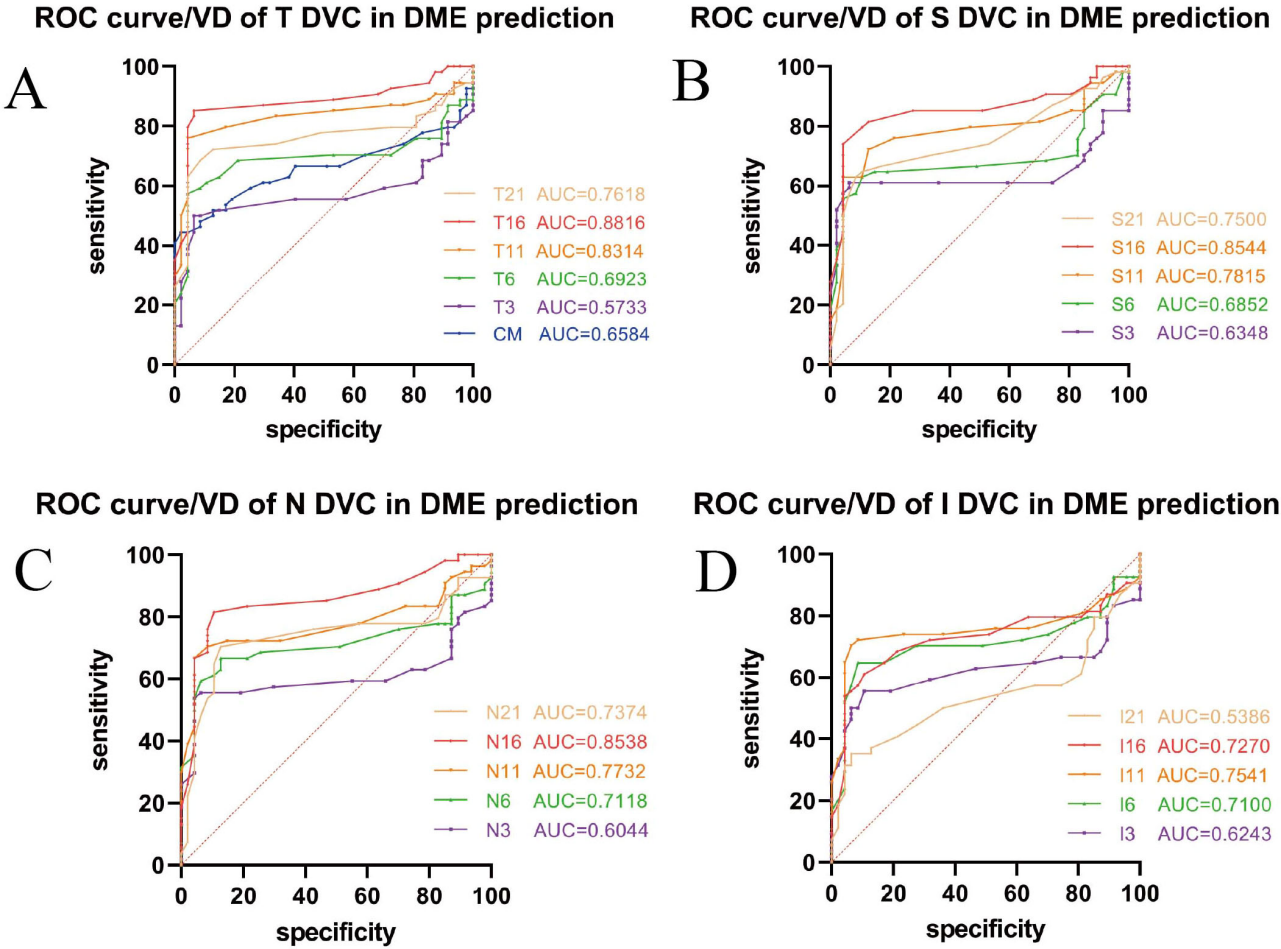
ROC curve analysis of DVC VD in patients with NPDR and DME. **(A)** Temporal quadrant, **(B)** Superior quadrant, **(C)** Nasal quadrant, and **(D)** Inferior quadrant. ROC, receiver operating characteristic; SVC, superficial vascular complex; VD, vascular density; NPDR, non-proliferative diabetic retinopathy; DME, diabetic macular edema.

ROC analysis revealed DME correlations (AUC>0.7) in 13 DVC regions, with particularly robust associations (AUC>0.8) in T11, T16, S16 and N16. These findings indicate that DVC vascular alterations may serve as reliable biomarkers for DME. Detailed results are presented in **Figure 4**, **Table 4**.

## 4 Discussion

This study elucidates vascular pathophysiological changes during three critical phases of DR progression. First, the nasal SVC exhibited significant VD reduction at N11, N16, and N21 in DM patients, while other regions remained unaffected, suggesting the nasal retina may represent the early affected area in DR. Second, during the NPDR stage, DME presence was associated with significant VD increases in peripheral regions (≥11mm) of both SVC and DVC, while the parafoveal region (3mm) showed no notable changes, indicating a strong correlation between DME and peripheral retinal vascular pathophysiology. Third, during DM and DR stages, extramacular DVC showed no significant VD changes, whereas DME exhibited marked VD increases, suggesting DVC possesses substantial compensatory blood flow capacity that becomes disrupted during DME. Notably, ROC analysis identified superior correlations between peripheral DVC regions (T11/T16/S16/N16) and DME development.

Of note, the present study identified a non-linear dynamic pattern of SVC VD changes during diabetic retinopathy progression. Specifically, SVC VD gradually decreased from the pure DM stage to the NPDR stage and increased significantly in the DME group, indicating that single SVC VD measurements are insufficient for accurate DR staging. In this context, simple correlation analyses between individual SVC VD values and DME onset cannot fully reflect the clinical characteristics of disease progression. Beyond SVC alterations, the current findings also underline the distinct role of the DVC in DME pathogenesis. Unlike the progressive fluctuating changes of the SVC, the DVC remained relatively stable during the DM and NPDR stages and displayed obvious VD elevation only after DME developed, suggesting that the DVC maintains a compensatory perfusion state that is disrupted upon macular edema formation. Consistent with the ROC results, peripheral DVC parameters showed favorable predictive performance for DME occurrence, indicating that region-specific DVC vascular features can help identify patients at high risk of DME. Rather than relying on independent VD values for DR staging, this study focused on characterizing stage-dependent and regional heterogeneous microvascular impairments of both SVC and DVC using ultra-widefield SS-OCTA. The VD elevation in both SVC and DVC observed in DME eyes may be explained by microvascular compensatory remodeling, inflammatory hyperperfusion, and secondary capillary dilatation induced by macular edema (3, 15, 16), rather than the recovery of microvascular injury. Overall, combined analysis of hemodynamic changes in regional superficial vascular complex and peripheral deep vascular complex is more conducive to screening relevant risk factors for diabetic macular edema than relying on single vascular density indicators.

Previous studies have reported that nasal retinal vessels are 25% smaller than temporal vessels, resulting in 2–3 times lower VD, rendering the nasal retina more vulnerable to pathological changes (17–20). Previous research also found that microaneurysm-like changes first appear in the nasal retina during DR progression, indicating that the nasal side is the early region affected by DR (21). The early appearance of microaneurysms in the nasal retina may be attributed to its proximity to the central retinal artery, resulting in higher perfusion pressure, whereas the temporal retinal vessels are more distant with lower perfusion pressure, making microaneurysms less likely to form (22). However, as the disease progresses, the temporal retina is more prone to developing non-perfusion areas, which may be attributed to the longer vascular course and lower perfusion pressure in the temporal retina (23, 24). This suggests that in the preclinical stage of DR, we need to focus on the pathophysiological changes of the nasal retinal microvasculature, while in the mild to moderate DR stage, we should pay more attention to the hypoperfusion changes in the temporal retina.

The SVC encompasses the internal limiting membrane to part of the inner plexiform layer, while the DVC includes the remaining portion of the inner plexiform layer to the outer plexiform layer (14). Thus, the SVC is primarily supplied by the central retinal artery system, whereas the DVC may also be influenced by choroidal blood supply in addition to the central retinal artery system (25). In the mild to moderate stages of DR, the SVC, due to its single blood supply, shows a significant decrease in VD, while the DVC, with its dual blood supply, exhibits no notable change in VD (26, 27). These findings are consistent with the results shown in **Figures 1**, **2** of our study. During this stage, intraocular VEGF release is insufficient to induce an increase in VD. However, by the DME stage, intraocular VEGF is released in large quantities, leading to a significant increase in VD in both the SVC and DVC (28, 29). Notably, the VD of the DVC, influenced by dual blood supply systems, becomes more pronounced, exhibiting a stronger correlation with DME. This aligns with the results shown in **Figure 4**. Concurrently, the heightened metabolic activity of photoreceptors leads to increased reactive oxygen species (ROS), rendering DVC more vulnerable. Impaired DVC blood flow, in turn, exacerbates ROS formation, creating a vicious cycle.

The above research can help us focus on changes in nasal retinal blood flow in clinical practice to monitor alterations in retinal blood flow before the onset of DR and explore earlier prevention and treatment methods for DR. Further investigation into the pathophysiological relationship between DVC VD and the development of DME may provide a new pathway to deepen our understanding of DME. Studies on the correlation between peripheral retinal blood flow changes and DME progression, as well as intraocular VEGF levels, may help elucidate the detailed progression of DME. This study also has limitations, as its conclusions only indicate associations rather than causality. Our study population was drawn from a single medical center, which may introduce selection bias and limit the generalizability of the findings. The conclusions of this study are only applicable to the NPDR stage, and further research is still needed for VD changes in the PDR stage. Specific blood pressure values were not recorded, and relevant comparative analyses were therefore unavailable. As this is a retrospective cross-sectional study, it can only reflect intergroup differences in retinal vascular indicators at a single time point, and cannot clarify the sequential progression of lesions or confirm definite causal relationships. Future prospective studies, particularly randomized controlled trials, are needed to validate our results.

## 5 Conclusions

jIn conclusion, this study highlights the nasal retina as the early affected region in DR, with significant VD reductions observed in the nasal SVC during early stages. The peripheral retinal vasculature shows a strong correlation with DME, particularly in the DVC, which exhibits compensatory blood flow capacity until disrupted by DME. Peripheral DVC vascular parameters are closely correlated with the presence of DME and can help distinguish patients with DME. These findings suggest that monitoring nasal retinal blood flow may aid in early DR detection, while further exploration of peripheral DVC pathophysiology could enhance DME understanding. However, prospective studies are needed to establish causality and validate these associations.

## Data Availability

The original contributions presented in the study are included in the article/supplementary material. Further inquiries can be directed to the corresponding authors.

## Glossary

AL: Axial Length
ANOVA: Analysis of Variance
AUC: Area Under the Curve
BCVA: Best-Corrected Visual Acuity
CM: Central Macula
DME: Diabetic Macular Edema
DM: Diabetes Mellitus
DR: Diabetic Retinopathy
DVC: Deep Vascular Complex
SVC: Superficial Vascular Complex
ETDRS: Early Treatment Diabetic Retinopathy Study
FA: Fluorescein Angiography
FAZ: Foveal Avascular Zone
FBG: Fasting Blood Glucose
HbA1c: Glycated Hemoglobin
IOP: Intraocular Pressure
LogMAR: Logarithm of the Minimum Angle of Resolution
NC: Normal Controls
NPDR: Non-Proliferative Diabetic Retinopathy
OCTA: Optical Coherence Tomography Angiography
PDR: Proliferative Diabetic Retinopathy
ROC: Receiver Operating Characteristic
ROS: Reactive Oxygen Species
SD: Standard Deviation
SS-OCTA: Swept-Source Optical Coherence Tomography Angiography
VD: Vascular Density
VEGF: Vascular Endothelial Growth Factor
